# Molecular Pathogenesis of Ischemic and Hemorrhagic Strokes: Background and Therapeutic Approaches

**DOI:** 10.3390/ijms25126297

**Published:** 2024-06-07

**Authors:** Carlo Domenico Maida, Rosario Luca Norrito, Salvatore Rizzica, Marco Mazzola, Elisa Rita Scarantino, Antonino Tuttolomondo

**Affiliations:** 1 Department of Internal Medicine, S. Elia Hospital, 93100 Caltanissetta, Italy; salvatore.rizzica@gmail.com; 2 Molecular and Clinical Medicine Ph.D. Programme, University of Palermo, 90133 Palermo, Italy; 3 U.O.C di Medicina Interna con Stroke Care, Dipartimento di Promozione della Salute, Materno-Infantile, di Medicina Interna e Specialistica di Eccellenza “G. D’Alessandro”, University of Palermo, 90133 Palermo, Italy; rosario94.norrito@gmail.com (R.L.N.); mazzolam94@gmail.com (M.M.); bruno.tuttolomondo@unipa.it (A.T.); 4 Division of Geriatric and Intensive Care Medicine, Azienda Ospedaliera Universitaria Careggi, University of Florence, 50134 Florence, Italy; elisarita.scarantino@unifi.it

**Keywords:** stroke, ischemic, hemorrhagic, pathophysiology, long non-coding RNAs, microRNAs, stroke therapeutics

## Abstract

Stroke represents one of the neurological diseases most responsible for death and permanent disability in the world. Different factors, such as thrombus, emboli and atherosclerosis, take part in the intricate pathophysiology of stroke. Comprehending the molecular processes involved in this mechanism is crucial to developing new, specific and efficient treatments. Some common mechanisms are excitotoxicity and calcium overload, oxidative stress and neuroinflammation. Furthermore, non-coding RNAs (ncRNAs) are critical in pathophysiology and recovery after cerebral ischemia. ncRNAs, particularly microRNAs, and long non-coding RNAs (lncRNAs) are essential for angiogenesis and neuroprotection, and they have been suggested to be therapeutic, diagnostic and prognostic tools in cerebrovascular diseases, including stroke. This review summarizes the intricate molecular mechanisms underlying ischemic and hemorrhagic stroke and delves into the function of miRNAs in the development of brain damage. Furthermore, we will analyze new perspectives on treatment based on molecular mechanisms in addition to traditional stroke therapies.

## 1. Introduction 

Stroke is classically characterized as a neurological deficit, attributed to an acute focal injury of the central nervous system (CNS) by a vascular cause. It is the third leading cause of mortality in the Western world and is the most common cause of persistent disability. Worldwide, its incidence is 15 million people per year, its mortality is around 5 million people per year and another 5 million remain permanently disabled [1,2,3,4,5]. One risk factor is ethnicity: for example, in the United States, the risk of the disease is greater in Hispanic and black people than in Caucasians, as reported by numerous studies [1]. Men are affected more than women: in fact, the incidence of stroke in men is approximately 62.8 per 100,000, while women have an incidence of stroke of 59 per 100,000. However, these data only concern young subjects; the American Heart Association recently demonstrated that women over 75 are more affected than men of the same age [2].

Furthermore, although the probability of stroke is directly proportional to age, and almost three-quarters of all strokes occur in patients over the age of 64 [1], stroke is not a pathology purely affecting the elderly; in fact, one-third of stroke cases occur in younger people.

Regarding the pathophysiology, we can identify two principal categories of stroke that are utterly different: hemorrhage and ischemia. Hemorrhage is represented by blood in the cerebral parenchyma, which can accumulate and press on the adjacent parenchyma.

In contrast, it is called ischemia when blood flow is insufficient and unable to satisfy the brain tissue’s needs for oxygen and nutrients. Cerebral ischemia is caused by a disruption of blood flow to the brain, and accounts for nearly nine out of ten cases of all strokes [6,7]. The TOAST classification distinguishes five different subtypes [8]: (1) large atherosclerosis of the arteries (LAAS); (2) cardioembolic infarction (CEI); (3) lacunar infarction (LAC); (4) stroke of other determined etiology (ODE); and (5) stroke of undetermined etiology (UDE). Most strokes of ischemic origin are caused by embolisms of cardiac origin and by atherosclerosis affecting the large artery.

A small or large artery thrombus provokes approximately 45% of ischemic strokes [8,9], whereas cardioembolic stroke accounts for 14–30% of all strokes [10,11]. Lacunar stroke is another important subtype (15–25% of all ischemic strokes) [12,13]. Therefore, cardiac embolism, the occlusion of small vessels and the atherosclerosis of the cerebral circulation can be the cause of ischemic stroke. Cardioembolic stroke is essential to know about because it provokes the most severe strokes [14], and, secondarily, although there are many therapies for dyslipidemia and arterial hypertension, embolism with cardiac origin represents a rising source of stroke in wealthy nations, for example, Canada [15]. 

Ischemic stroke and hemorrhagic stroke share different pathophysiological mechanisms, such as oxidative stress and inflammation; among the other most important mechanisms in ischemic stroke are calcium overload and excitotoxicity [16,17].

Given the diversity of the pathophysiological mechanisms involved, therapeutic strategies continually evolve in search of potential pharmacological targets. Therefore, this review focuses on the pathophysiology, molecular basis and potential therapeutic strategies for stroke, both those consolidated and newly evolving.

## 2. Ischemic Stroke Pathophysiology 

As previously described, ischemic stroke occurs when the cerebral arteries occlude due to a travelling embolus, such as a cardiogenic embolus, an artery-to-artery embolus or a vascular stenosis of the artery itself. Cerebral blood flow is interrupted, resulting in functional and neurological damage. Most strokes of ischemic origin are caused by atherosclerosis, affecting the large artery, and by embolism with cardiac genesis.

**Figure 1:**
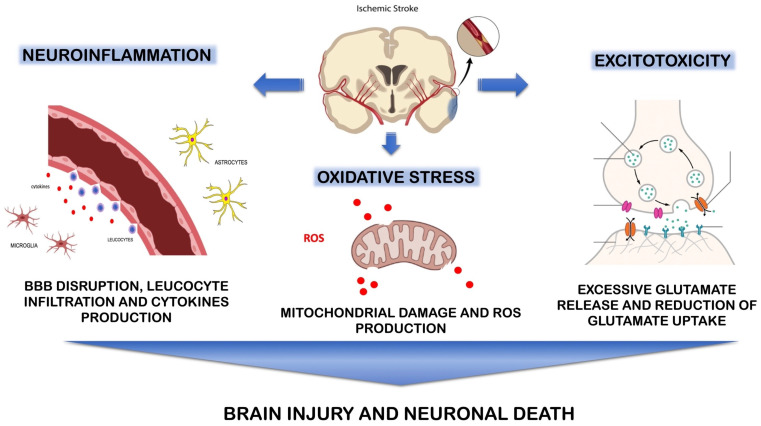
A summary of the pathophysiology involved in ischemic stroke. Excitotoxicity, neuroinflammation and oxidative stress, which manly involves autophagy, apoptosis and necroptosis in ischemic stroke. BBB: blood–brain barrier; ROS: reactive oxygen species.

Ischemic and hemorrhagic strokes manifest in neurological deficits due to various pathophysiologic mechanisms (Figure 1). 

### 2.1. Atherothrombotic Stroke

Atherothrombotic stroke is the most common. Atherosclerosis is described as a reduction in caliber or a hardening of the arteries, and usually affects medium and large arteries. The process begins with damage to the internal coating of an artery (the endothelium). The damage can occur secondly from physical stress, such as arterial hypertension. Damage to the arteries can also be caused by excess cholesterol in the blood or high blood sugar, which is consequently inflammation-mediated by the immune system. Grassi, cholesterol, platelets, cellular debris and calcium gather in the walls of damaged arteries, stimulating the creation and accumulation of other types of cells. The plaque accumulates fat inside with connective tissue around it [18]. The resulting deposits are highly thrombogenic because they hinder blood flow and exercise high cutting stresses on the vessel wall [19]. The plaque thickens the artery wall, narrowing the vessel. Blood flow is decreased by the narrowing, reducing the oxygen supply to the part of the body that the artery serves. One of the principal causes of ischemic stroke is atherosclerosis of the carotid artery, with artery-to-artery embolism being the central stroke mechanism in patients with atherosclerosis of the carotid artery [20]. Another biophysical mechanism of obstruction is the development of a local thrombus within a cerebral artery [21]. As a response to vessel injury or atherosclerotic lesions, there is thrombosis, or the formation of a blood clot [22]. The coagulation cascade is activated, resulting in the aggregation of platelets and the transformation of prothrombin into a fibrin clot.

### 2.2. Embolic Stroke

In contrast to a thrombus, an embolus represents a travelling or migrating source of obstruction, thus originating from a distant site. An embolus can be arterial, cardiac, from the peripheral circulation, aortic, or from an unknown source. Cardiac embolism represents an increasing percentage of cerebral ischemia, and it will probably rise predominantly in the future. This kind of stroke is caused by an occlusion of a cerebral artery from an embolus formed in the heart. The most severe type of ischemic stroke is considered to be cardioembolic stroke, and emboli larger in size correspond with higher severity in outcome and injury. From all possible cardiac origins, there are different risks of causing an ischemic stroke; in fact, some of them have a medium risk of provoking embolism, and others have a high risk instead. It is necessary to identify at least one cardiac cause of embolism to establish that cardioembolism produced cerebral ischemia. The most common cause of cardioembolic strokes seems to be the low cardiac output and blood stasis associated with atrial fibrillation. Other high-risk cardiac conditions include valvular heart disease, acute myocardial infarction, bacterial endocarditis and dilated cardiomyopathy [23]. Interestingly, a hypercoagulable state has proven to be associated with the novel severe acute respiratory syndrome coronavirus 2 (SARS-CoV-2), potentially increasing the risk of embolic strokes in COVID-19 patients [24]. Furthermore, an important, although less common, primary cause of stroke is blood and clotting disorders. This heterogeneous group of pathologies should be suspected as an underlying cause of brain damage in young patients under 45 years of age, and especially those with a history of cryptogenic stroke and coagulation dysfunction [25]. Blood disorders associated with arterial cerebral infarction include the following:

- Polycythemia vera;
- Essential thrombocytosis;
- Heparin-induced thrombocytopenia;
- Protein C or S deficiency, acquired or congenital;
- Prothrombin gene mutation;
- Factor V Leiden (resistance to activated protein C);
- Antithrombin III deficiency;
- Antiphospholipid syndrome;
- Hyperhomocysteinemia;
- Thrombotic thrombocytopenic purpura (TTP).

In particular, the Factor V Leiden and prothrombin 20210 mutations can lead to thrombosis of the cerebral and deep venous systems, with the possible development of paradoxical emboli. Furthermore, several disorders that share an inflammatory and infectious origin, including pneumonia, urinary tract infections, Crohn’s disease, ulcerative colitis, HIV/AIDS and cancers, may promote active thrombosis and embolism by raising fibrinogen, C-reactive protein and coagulation factors VII and VIII. 

## 3. Molecular Mechanisms of Ischemic Stroke Pathophysiology

The onset of ischemia is followed by a cascade of events producing or activating different components, disrupting the homeostatic balance within the brain and leading to cellular death. Among these, excitotoxicity, oxidative stress and the inflammatory response play vital roles [26,27,28,29].

### 3.1. Excitotoxicity and Calcium Overload

A cascade of events producing or activating different components follows the onset of ischemia, which leads to cellular death. After cell death, stressors such as organelle swelling, plasma membrane disruption and cellular content leakage are responsible for neuronal damage [30]. The fundamental processes that contributes most to the genesis of the pathological picture are inflammation, the secretion of excitatory amino acids, the increase in intracellular calcium levels and the production of prostaglandins, leukotrienes and reactive oxygen species, and these are potential targets of interest in therapeutic research [31]. In the early stages of ischemic damage, excitotoxicity and calcium accumulation are closely related. The consequences of ischemia are energy deficit and the failure of the ion pumps and reuptake mechanisms, whereby the excitatory amino acids, mainly represented by glutamate, are released and accumulate in the neuronal extracellular space. The increased glutamate concentration and prolonged activation of NMDA and AMPA glutamate receptors lead to the influx and increase in intracellular calcium concentration [32].

Significant shifts in ion homeostasis are secondary to AMPA receptor overstimulation, accounting for the increased concentrations of intracellular sodium and chloride [33]. This ion imbalance triggers a water influx into the neurons, leading to cell lysis and tissue edema [34].

Furthermore, increased intracellular calcium concentration facilitates the release of calcium from mitochondria and other cellular stores, resulting in mitochondrial dysfunction [35] and underlining the complex role of calcium in excitotoxicity. As well as glutamate receptors, ion channels and pumps involved in calcium dysregulation during ischemia, such as acid-sensing ion channels and the Na/Ca^2+^ exchanger, are also considered potential targets in ischemic stroke injury [36]. Mitochondrial dysfunction leads to swelling, wall permeability and membrane collapse, thus triggering apoptotic and oxidative stress [37].

### 3.2. Oxidative Stress

Brain ischemia and, to a more significant extent, brain reperfusion are also associated with producing free oxygen radicals. The significantly increased calcium concentration activates some calcium-dependent enzymes, such as nitric oxide synthase (NOS), which leads to cell damage following the formation of reactive oxygen species (ROS), such as peroxynitrite (ONOO-) [38]. Mitochondria have long been considered a primary source of ROS during ischemia and play a primary role in the generation of ROS during secondary reperfusion injury.

The swelling and collapse of the mitochondria due to mitochondrial dysfunction involves activating pro-apoptotic cascades and releasing free oxygen radicals [39]. A key contributor to ROS production is the enzyme NADPH oxidase, which has recently been shown to generate the majority of superoxide anions in ischemia and NMDA receptor activation compared to mitochondria [40].

### 3.3. Neuroinflammation

After ischemic stroke, the brain insult results in apoptosis and necrosis, each of which drive an inflammatory response; this response is called neuroinflammation, and is characterized by the participation of numerous cytotypes, first with the activation of resident glial cells and then with the infiltration of leukocytes, monocytes and other immune cells in the brain, and the releasing of inflammatory agents. After infiltration into the lesion, dynamic immune cascades occur almost simultaneously to induce beneficial and detrimental effects after stroke onset and continue in the later phases. This immune system is summarized in Table 1. 

#### 3.3.1. Roles of Cytokines in Cerebral Ischemia

Cytokines are immunomodulating agents that play a significant role in cell activation, proliferation and differentiation. Almost every nucleated cell can produce cytokines to modulate the interaction of immune cells such as T cells, B cells and monocytes/macrophages, thereby arranging immune responses [41,42]. Many processes in the brain involve proinflammatory cytokines that can trigger neurons, glia and endothelial cells directly. Because of this complex and multiphasic pathway, they may induce further damage or increase cellular viability.

##### TNF-α

One of the proteins most involved in an inflammatory and immune process is TNF-α, in the past called differentiation factor or cachectin.

This cytokine can be produced by monocytes, mast cells, T cells, macrophages, keratinocytes, neutrophils and fibroblasts. TNF-α has two different forms: a soluble one, which is its biologically active form (sTNF-α) made by the tumor necrosis factor-alpha-converting enzyme (TACE), and a transmembrane one (tmTNF-α), which modulates inflammation locally via cell–cell interactions [43]. The soluble form operates systemically by promoting macrophages’ cytotoxic and phagocytic action and stimulating the production of other cytokines, such as IL-6 and IL-1. TNF works not only systemically but also locally in the brain. TNF-α acts through receptors (TNFR-1 and TNFR-2), which have a different affinity for TNF-α and the degree of glycosylation. sTNF-α is correlated with TNFR1, while tmTNF-α is with TNFR-2 and TNFR-1. Growing evidence suggests that TNF-α plays a crucial role in the pathophysiology of stroke and provokes both a neuroprotective and a neurotoxic result in the ischemic brain [41,42]. Proinflammatory mediators, such as interleukin-1 (IL-1), TNF-α and IL-6, contribute to developing harmful post-ischemic inflammation in the brain [33,34,35]. Increasing evidence from studies [32,33], using both preclinical animal models and human samples collected from patients affected by ischemic stroke, suggests that TNF-α, a pleiotropic and powerful proinflammatory cytokine, is involved in the development of injuries in the ischemic brain.

Furthermore, this cytokine is overexpressed in the brain after both transient [44] and permanent [45] middle cerebral artery occlusion [MCAO]. TNF-α is one of the first cytokines to emerge in the context of the inflammatory response to ischemic brain injury and contributes to stimulating the cascade of other inflammatory components in both blood serum and the cerebrospinal fluid [46,47]. Indeed, TNF-αs’ appearance in the brain following post-ischemic damage is early because it has an initial peak in the first hours [1–3 h] and a second one after more than 24–36 h [46,47]. In the literature, some reports indicate that TNF-α represents a valuable indicator for evaluating prognosis and an accurate parameter for determining the beginning of the inflammatory response [48]. In stroke patients, as soon as 6–12 h after the onset of symptoms [49], a rising concentration of TNF-α can be seen. An increase in TNF-α concentration within 24 and 48 h following a stroke has also been shown; the slight decrease, which occurs within 72 and 144 h after a stroke, correlates with clinical improvement in patients during the acute phase of ischemic stroke [50]. Furthermore, TNF-α is also involved in the neuroprotective process against ischemic brain damage [51,52]. This cytokine seems to play a bivalent role in the brain’s inflammatory responses that follow ischemia, because it plays an immunosuppressive role during the chronic phase and a proinflammatory role throughout the acute phase of the inflammatory response in the CNS.

##### IL-1β

The IL-1 family includes at least three proteins, IL-1α, IL-1β and IL-1ra, that are the products of separate genes sharing a significant homology and are implicated in the pathogenesis of many human diseases, including stroke. IL-1β is a principal proinflammatory and immunoregulatory cytokine able to influence almost all cell types. IL-1β seems to be the central IL-1 agonist induced in the brain after responding to local insults (e.g., trauma or stroke) or systemic ones (e.g., infection or injury) within one hour during experimental cerebral ischemic brain injury. IL-1β is produced following the formation of an inflammasome, such as a monocyte or macrophage/microglia [53]. After ischemic stroke, IL-1β can turn on the nuclear factor (NF)-κB through the activation of TLRs; after this process, NF-κB can transactivate genes correlated with cytokines, chemokines and other proinflammatory agents [54]. 

After ischemic stroke, the microglia will be switched to the proinflammatory phenotype called M1, able to express IL-1β, which is a proinflammatory cytokine with neurotoxic effects. Additionally, IL-1β can interact with the endothelium and increase leukocyte adherence, promoting edema formation [55]. IL-1β knockout mice have significantly decreased brain injury induced by MCAO [56]. Furthermore, brain injury increased when IL-1β was administered to rats [57]. Multiple studies report that inhibiting IL-1 receptor 1, which binds to both IL-1α and IL-1β and is detected in various cytotypes, reduces the area of the brain damaged by ischemia, preserving neurological functions [58]. We must, therefore, consider IL-1βa a crucial factor in ischemic brain damage. IL-1β, when bound to its receptor, the IL-1 receptor (IL-1R), causes an IL-1R-dependent increase in NF-κB pathways.

However, if the levels of IL-1β are increased above a specific cutoff, it can promote the expression of the IL-1 receptor antagonist [IL-1Ra]. This balance between IL-1β and its antagonist, IL-1Ra, is more critical than just IL-1β, because of its global effect and role [59]. Thus, this balance might be a good predictor for a patient’s outcome following an ischemic stroke. However, just a few clinical studies have used their level as stroke biomarkers. IL-1β levels were mainly correlated with poor long-term functional outcomes in the study [60]; on the other hand, IL-1Ra levels seemed to predict post-stroke infection development [61].

##### IL-6

Various cytotypes, including microglial cells, leukocytes, astrocytes and endothelial cells, can produce IL-6 in response to brain injury. It stimulates hepatocytes to synthesize acute phase proteins (APPs), primarily fibrinogen and CRP (C-reactive protein). IL-6 activates APPs and involves the phosphorylation of the NF-IL-6 transcription factor, which can enhance the transcription of numerous genes [62]. The production of IL-6 requires IL-1 and TNF, which stimulate endothelial cells, fibroblasts and keratinocytes, thereby increasing the expression of IL-6. IL-6 is a proinflammatory cytokine with several crucial beneficial and harmful functions to CNS cells. Various other molecules, such as IL-1, interleukine-4 (IL-4), prostaglandins and TNF-α, can trigger and modulate the production of this cytokine, suggesting that the process of expression of various cytokines is intricately linked to the inflammatory cascade. This complexity presents a fascinating challenge for further research in this field.

In recent years, many pieces of research have been made attempts to explain the role of interleukins in the etiology and development of stroke. Although IL-6 is a cytokine with a proinflammatory role, it has been proposed that it plays an essential function in cerebral ischemia, not only as a mediator of the inflammatory development in the acute stage of stroke, but also as a neurotrophic element during the late phase of the progress of cerebral ischemia [63]. It has been verified that IL-6 is known as an essential inflammatory marker in stroke; several studies proved a meaningful increase in the concentration of IL-6 in serum, which took place within a few hours following the onset of ischemia and lasted for up to 90 days after the stroke [64]. Thus, these studies show that IL-6 concentration increases during an ischemic stroke, while in physiological conditions, IL-6 cerebral expression is modest [64].

Conversely, the administration of recombinant human IL-6 has shown significant reductions in ischemic damage in a rat model of stroke [64]. Moreover, Sotgiu et al. [50] have reported a negative correlation between the measure of cerebral infarction and the level of IL-6. From this, the authors have concluded that in the intricate network of inflammation that occurs during an ischemic stroke, IL-6 is not a neurotoxic factor but a neuroprotective one. This compelling evidence suggests a potential bivalent role of IL-6 in the ischemic brain, inspiring further exploration into its therapeutic applications.

##### IFN-γ

The IFN group cytokines can be divided into two types. Type I IFNs represent the largest class and comprise the IFN-α, -β, -ε, -κ and -ω, which share remarkable sequence homology and are produced by most cytotypes. IFN-γ is a unique member of the type II IFN and has a crucial role in stimulating and modulating an array of immune responses [65]. Mostly monocytes, macrophages, natural killer [NK] cells, T cells, dendritic cells and B-lymphocytes secrete IFN-γ. It is a significant regulator of immune function and offers a robust first-line defense against invading pathogens.

Furthermore, IFN-γ has many biological actions, including regulating multiple aspects of the immune responses and promoting antigen presentation via upregulating class I and class II major histocompatibility complex [MHC] molecules on the surface of macrophages and T cells. The heterodimeric receptor (IFN-γR) on the cell’s surface mediates the cellular response to IFN- γ, activating downstream signal transduction cascades, ultimately regulating gene expression. IFN-γ, when bound to its related receptor, can switch a variety of downstream signaling pathways on, in particular, the Janus kinase (JAK)/signal transducer and activator of transcription (STAT) [66]. The potential influence of IFN-γ on atherogenesis and ischemic stroke is a compelling area of research, with a growing body of evidence reporting its greater expression in atherosclerotic lesions [67]. This suggests its crucial function in the process of atherogenesis and its potential role in the pathogenesis of ischemic stroke, underscoring the need for further investigation.

Following an ischemic brain insult, MHC class II-specific CD4 cells will be activated and may effortlessly penetrate the CNS through the blood–brain barrier [BBB] [68]. Therefore, microglia have the chance to retain and further arouse CD4 cells already primed to differentiate into either T helper 1 [TH1] cells, able to produce proinflammatory cytokines such as IL-6, IFN-γ and TNF-α, or into T helper 2 [TH2] cells, producing cytokines capable of supporting antibody-mediated responses [IL-4, IL-5, IL-10, and lL-13] [69]. The role of IFN-γ in the polarization of microglia is a crucial aspect of its function. TH1 cells produce proinflammatory IFN-γ cytokines that can turn microglia into M1 phenotypes; this cytotype is responsible for a proinflammatory reaction and can produce oxidative metabolites and proinflammatory cytokines, highlighting the significant impact of IFN-γ on the immune response.

##### Anti-Inflammatory Cytokines

The unevenness between inflammatory and anti-inflammatory responses seems critical in brain damage after ischemia [70].

IL-10 is an anti-inflammatory cytokine and is encoded on chromosome 1 by the IL10 gene; monocytes are the main source of its production and, to a lesser extent, it is also produced by mast cells, TH2 lymphocytes, regulatory T cells (CD4 CD25 Foxp3) and by a specific subset of activated B and T cells [70]. The fundamental role of IL-10 in the pathogenesis of stroke is suppressing the excessive production of cytokines that promote inflammation. This emerged in a study that demonstrated a potential role for IL-10 in reducing the infarct area in normal mice [70]; similarly, larger lesions are found after MCAO in IL-10-deficient mice [71].

TGF-β is a pleiotropic growth factor with three isoforms that bind the same receptors (TGF-β1, TGF-β2 and TGF-β3). It is involved in neuronal support and the repair of brain tissue damage that occurs subsequently to a brain injury.

Studies have shown [72] that TGF-β exerts a suppressive action against neutrophils and astrocytes, capable of producing inflammatory cytokines with a harmful effect during the inflammatory response to cerebral ischemic injury. After ischemic stroke, TGF-β, provided by activated M2 phenotype macrophages, is anti-inflammatory and contributes to healing after brain injury [73]. TGF-β reduces the potentially damaging effects associated with activated microglia by inhibiting microglial activation. Therefore, TGF-β appears to have a neuroprotective and anti-inflammatory role in ischemic stroke.

IL-4, a cytokine that can regulate various immune and inflammatory responses, plays a vital role during TH2 cell differentiation [74]. It can also polarize macrophages/microglia toward the anti-inflammatory M2 phenotype [75]. M2 macrophages/microglia express anti-inflammatory mediators and produce various neurotrophic factors that support the resolution of inflammation, mediated by an increased trophic input, and the enhancement of both the phagocytosis and proteolysis of dead, diseased cells/proteins. This ultimately promotes tissue restoration [76]. Therefore, IL-4 may have a neuroprotective action, encouraging tissue repair and potentially playing a therapeutic role.

#### 3.3.2. Recruitment of Inflammatory Cells in Ischemic Brain Injury

##### Microglia

Microglia are part of the resident innate immune cells of the brain and represent 5–20% of the glial population. This cytotype is activated after ischemic stroke, and morphological and phenotypical changes can be observed [77]. 

Among the first cells involved in the response to brain injury are the resident microglial cells, which are activated within a few minutes of the ischemic brain insult and tend to increase their number in the following days, reaching their maximum peak approximately ten days after the brain ischemia focal transient [78]. At the same time, macrophages from the bloodstream arrive with a physiological delay. They begin to appear in damaged brain tissue on the fourth day, reaching a peak on the seventh day, and then decline [78].

After ischemic brain injury, microglia become activated, branching is retracted and they assume an amoeboid morphology identical to activated macrophages. These changes contribute to the acquisition of macrophage-like abilities, including phagocytosis, cytokine production, antigen presentation and the production of matrix metalloproteinases that can damage the BBB, increasing its permeability. All this can favor an early infiltration of circulating leukocytes into the brain, consequently exposing it to systemic inflammatory agents that worsen ischemic damage.

Activated microglia have two phenotypes: classically activated [M1] and alternatively activated [M2] [79]. The M1 microglia play a proinflammatory role and thus secrete cytokines and oxidative metabolites such as IL-1β, TNF, IL-6 and nitric oxide [80]; on the contrary, M2 microglia contribute to recovery after brain injury. This one also expresses anti-inflammatory mediators, such as IL-10 and IL-4, producing neurotrophic factors that prevent inflammation and promote recovery.

During ischemic stroke, the M2 phenotype is predominant in local microglia and newly recruited macrophages at earlier phases. The M1 phenotype increases gradually in peri-infarct areas. Thus, neurons in ischemic regions induce changes in the M2 phenotype in microglia and macrophages [80]. Reflecting on the opposite roles of microglia phenotypes in ischemic stroke, developing a therapeutic strategy to suppress the morphological modification and encourage the benefits of microglia is crucial.

##### Astrocytes

Like microglia, astrocytes are the resident cells of the brain with a fundamental role in maintaining homeostasis in the central nervous system. They actively modulate the ion and water balance, release neurotrophic agents and scavenging transmitters released during synapses and shuttle metabolites and waste products; astrocytes are also involved in BBB formation [81]. In normal physiological conditions, excessive glutamate is taken up by astrocytes from the extracellular space and converted to glutamine for new neuronal utilization. Still, during brain injury, the extent of damage to the astrocytes might negatively influence their ability to uptake glutamate [81]. After ischemia, cytokines produced by neurons and glial cells conduct the astrocyte’s reactivity hyperplasia. Astrocyte’s proliferation ends in the expression of inflammatory agents, such as monocyte chemotactic protein-1, IL-1β [82], glial fibrillary acidic protein [GFAP], nestin and vimentin, that can cause reactive gliosis and scar formation [83]. 

After a stroke, because of the failure of the Na^+^ and K^+^ pumps, astrocytes enlarge; this fact provokes high intracerebral pressure and less cerebral perfusion [84]. Reactive astrocytes liberate matrix metalloproteinase too, leading to matrix protein degradation [85]. Reactive astrocytes also result in inhibitory conditions by inducing ephrin-A5 at the lesion center, interfering with axonal sprouting [86].

##### Neutrophils

Aside from microglia and blood-derived macrophages, neutrophils are one of the most significant leukocytes infiltrating the ischemic brain. They come out early, between thirty minutes and a few hours following ischemic injury, reaching a peak within the first three days and gradually declining over time [87]. Further, neutrophil infiltration rises on day 1, spikes on day 3 and begins to decrease but is detectable through days 7 and 15 after cerebral ischemia. Neutrophils express different endothelial adhesion molecules within 15 min post-ischemia [88]. Neutrophils have already encompassed cerebral vessels after 6 to 8 h of the stroke and immediately start infiltration [89]. In the pathogenesis of cerebral ischemic damage, neutrophils play a complex role through several possible mechanisms, such as the reduction in cerebral blood flow [CBF] through the obstruction of the vessels or the secretion of vasoconstrictor agents and the increased production of the hydrolytic enzyme, ROS and proinflammatory molecules [90]. Furthermore, as is MMP-9, the matrix metalloproteinase most involved in brain damage secondary to ischemia, neutrophils are also involved in ischemic damage.

They are a protease able to degrade the basal lamina and encourage the disruption of the BBB after cerebral parenchymal damage, causing edema and the hemorrhagic transformation of ischemic stroke [91].

The infarct volume and functional deficits are proportional to the neutrophil increase [87]. On the other hand, the number of lymphocytes declines during ischemic stroke, and, hence, the neutrophil–lymphocyte ratio increases. This ratio is intimately related to infarct size and mortality [92].

##### Dendritic Cells (DCs)

DCs are professional antigen-presenting cells (APCs) that express MHC II to promote T cell activation [83]. However, DCs are usually not found in the brain parenchyma [93]. Their role is well documented in animal models of ischemic stroke. After inducing transient MCAO in mice, DCs accumulate in the ischemic hemisphere at 24 h.

A further typification of the DCs involved was carried out; from the periphery, the DCs moved to the central area of the infarct, while the resident DCs mainly occupied the penumbra [94]. In patients with acute ischemic stroke, a significantly reduced number of circulating myeloid DC precursors (mDCPs) and plasmacytoid DC precursors (pDCPs) were observed, a concentration that increased again after a few days. This transient decrease in circulating DCPs may be due to recruitment from the blood into the ischemic brain. Patients with lower DCPs show larger stroke lesion sizes and co-localizations of myeloid DCs with T cells around cerebral vessels in the infarct area. This may mean that antigen-mediated T cell activation and immune responses of long durations occurred in the infarcted area [95].

##### T lymphocytes

Unlike neutrophils, T lymphocytes play a crucial role in the later stages of ischemic brain injury. These leukocytes infiltrate the peripheral zone surrounding the lesion by day three, sparing the center and often in proximity to blood vessels. Their numbers increase on the third day, peak on the seventh day and decline during the next seven days [96]. The growing body of research is focused on unravelling the function of different T cell subtypes in the complex pathophysiology of ischemic stroke. 

Three main groups of T cells can be distinguished based on their function: cytotoxic T cells [CD8+], helper T cells [Th, CD4+] and regulatory T cells [Tregs]. Each subtype has distinct markers on the cell surface and unique cytokine secretion profiles, providing valuable insights into T cells’ diverse nature and function. Notably, studies on cerebral I/R in T cell-deficient mice have shown that CD4+ and CD8+ T cells play a crucial role in brain inflammatory and thrombogenic responses, brain damage and neurological symptomatology associated with experimental stroke by operating on a shared pathway [97].

Tregs, a significant CD4+ T-cell subtype, represent 10% of all CD4+ T cells and express CD25 on the surface and the transcription factor Foxp3. Researchers are exploring the potential of modulating the activity of these cells due to their neuroprotective activity.

Several studies [98,99] suggest that Treg lymphocytes and their main cytokine, IL-10, represent the main protective factors against inflammatory brain damage following an ischemic episode. They appear to act by counteracting the harmful production of inflammatory cytokines, such as IFN-γ and TNF-α, and by reducing the activation and infiltration of other immune cytotypes, such as microglia and leukocytes, which amplify the inflammatory reaction of the post-ischemic brain.

Moreover, the immunodepletion of Treg, mediated by the CD-25-specific antibody, was associated with more serious neurological symptomatology at day seven, after MCAO induced ischemia in mice [99] and exacerbated tissue loss. However, Treg cell therapy is already under study, including strategies such as isolation and purification, a period for ex vivo expansion, and the use of its anergic properties.

Populations of T lymphocytes also contain a small subset, γδ T cells, a unique and conserved population of lymphocytes that is about 5% of T cells. γδ T cells express a peerless T cell receptor [TCR], usually composed of α and β glycoprotein chains, made by one γ chain and one δ chain. Even this cytotype seems to be involved in the pathogenesis of ischemic stroke, as shown by experimental studies where it was observed to induce a significant decrease in infarct extent in TCR-γδ knockout mice; the same result was noticed in mice treated with the TCR-γδ-specific antibody [100]; this evidence may propose new prospective therapies to modulate the brain’s inflammatory response following ischemia. 

Inside the circulating CD4+ T cell population, there is a subset of cells lacking the costimulatory molecule CD28 on their surface. This unusual subset of helper cells is called CD4+CD28 null, a sparse population in most healthy subjects; indeed, it represents 0.1–2.5% of peripheral blood total CD4+ T-lymphocytes. Once activated, they are proinflammatory, producing large amounts of IFN-γ and TNF-α [101,102]. Furthermore, they have a harmful effect by expressing cytotoxic molecules usually produced by TCD8+ lymphocytes and NK cells, not by TCD4+ lymphocytes, such as granzyme A, granzyme B and perforin. Further essential properties of these cells are represented by the fact that they are immune to apoptotic cell death, unresponsive to suppression by natural regulatory T cells, and are also incapable of providing signals required for antibody production by B lymphocytes due to the lack of CD40 molecule expression caused by the absence of the CD28 receptor [103]. Nowik et al. [104] assessed whether these cells might play a pathogenic function during an ischemic stroke with an atherosclerotic background, showing that CD4+CD28 null lymphocytes are involved in processes able to increase the risk of acute ischemic stroke, rejecting that their appearance is just a consequence of stroke. The authors showed that the percentage of CD4+CD28 null lymphocytes was not significantly different between the two groups [a group in the acute phase of ischemic stroke and another one with no story of ischemic stroke, but with either hypertension or diabetes] but was significantly higher than in the controls. This difference may suggest a possible connection between these cells and the other risk factors in accelerating plaque destabilization and atherosclerotic development. Moreover, this study also proved that the intensity of neurological damage, evaluated by the presence of or increase in an ischemic lesion observed in a brain CT scan performed within 24 h from the stroke onset, is not associated with the percentage of lymphocytes. 

This finding contrasts with the hypothesis that an increase in these cells follows the cerebral ischemic insult, suggesting that their rise occurs before stroke onset. 

In another study conducted by Z.G. Nadareishvili et al. [105], the concentration of CD4+CD28 null lymphocytes was studied, and they evaluated whether there was an increase in patients who died after acute ischemic stroke or frequent stroke. In this study, we evaluated the percentage of CD4+CD28 null lymphocytes in peripheral blood in 106 patients during the first 48 h after the onset of symptoms, and a follow-up was conducted up to one year after the onset of stroke symptoms. The authors correlated a higher percentage of these cells with a higher risk of stroke recurrence or death, proposing that the increase in the number of CD4+CD28 null lymphocytes may represent a biomarker for recurrent stroke and death. Multiple factors could explain the results obtained, such as the fact that CD4+CD28 null cells can cause the first and recurrent strokes that damage the endothelium and brain tissue through inflammation, or perhaps this subset of T lymphocytes could be associated with defects of adaptive immunity and, consequently, considered an indicator of poor general health.

Tuttolomondo et al. performed a study [106] to examine the peripheral concentration of CD28 null cells in subjects with acute ischemic stroke classified into the TOAST subtype, and to evaluate their correlation with acute ischemic stroke clinical severity scores and their predictive function in the diagnosis of acute ischemic stroke, and in determining the diagnostic subtype. Ninety-eight subjects diagnosed with ischemic stroke were enrolled in the study; 66 hospitalized patients without a diagnosis of acute ischemic stroke were enrolled as controls. The authors highlighted that subjects with acute ischemic stroke had a significantly higher peripheral frequency of CD4+ cells and null CD28 cells than the control subjects. Furthermore, subjects with cardioembolic stroke had a significantly higher percentages of CD4+ cells and null CD28 cells than subjects with other TOAST subtypes. Finally, the degree of neurological deficit during the acute phase was correlated with the external percentage of null CD28 cells and some markers of stroke severity, such as the NIHSS and SSS score, used to evaluate the type. The results of this study hypothesize that a higher percentage of peripheral CD4+ CD28 null cells may be associated with deeper brain injury.

In humans, a dominant part of natural killer (NK) and T cell target recognition depends on the monitoring of human leukocyte antigen (HLA) class I molecules by killer immunoglobulin-like receptors (KIRs). Recent evidence [107] suggests T cells’ implication in the acute complication of atherosclerosis, implying a possible function of KIRs’ genetic background in regulating inflammatory cell entanglement during the acute cardiovascular event. Based on this, Tuttolomondo et al. performed a study [108] to increase the understanding of the immunological genetic background of acute ischemic stroke susceptibility in correlation with the frequency of the KIR genes and HLA alleles.

Between November 2013 and February 2016, 116 patients with acute ischemic stroke and 66 subjects without acute ischemic stroke were enrolled as healthy controls. Acute ischemic stroke patients and control patients were divided by genotype for the presence of KIR genes and the three major groups of KIR ligands, HLA-C1, HLA-C2 and HLA-Bw4, both HLA-B and HLA-A. A greater frequency of activation of “proinflammatory” KIR genes was highlighted in patients with ischemic stroke, and this could justify immunoinflammatory activation during the acute phase of the stroke. 

Indeed, the previous finding [106] of an increase in the percentage of CD28 null cells in the peripheral blood of subjects with ischemic stroke may also be secondary to the greater prevalence of KIR gene activators such as 2S2 and 2DS4.

The authors concluded that an increased degree of cytokine and cell-mediated (NK cells or T cell subsets such as CD4+CD28 null cells) inflammation could also depend on KIR genes, which are responsible for expressing activator KIR receptors on these inflammatory cytotypes.

##### B Cells

The protection of brain cells after ischemic stroke can also be provided by B cells, depending on IL-10. However, unlike T cells, B cells showed no improvement in reducing inflammation or brain injury when MCAO was induced in B cell-deficient mice [32]. μMT/B cell-deficient mice showed more extensive lesions and a higher mortality rate after 48 h of MCAO than wild-type mice. After 48 h, leukocytes such as neutrophils, T cells, microglia and macrophages producing IFN γ and TNF α were accumulated in the ischemic hemisphere of μMT mice. A transfer of IL-10 −/− B cells to μMT/mice did not show a reduction in the infarct area after MCAO, as B cells can restore the beneficial outcome, including the inhibition of the release of proinflammatory cytokines from T cells after stroke, through the production of IL-10 [109].

Similarly, the adoptive transfer of naïve B cells from wild-type mice to MCAO-induced mice generated smaller ischemic lesions at 3 and 7 days. The same experiment showed no protection with IL-10-deficient B cells. Using serial two-photon volumetric tomography of the whole brain, a shift in B cells was observed in areas distant even from the lesion area, such as the cerebral cortex, the dentate gyrus, the olfactory areas and the hypothalamus, where they govern motor and cognitive skills and promote long-term recovery. These observations show that B cell depletion impacted neurogenesis and cognitive functions post-stroke [110]. Another study showed that B cells proceeded with a delayed infiltration into the lesion approximately seven weeks after onset. During that period, B cells were closely associated with T cells and CD11c-expressing cells, potentially DCs, for antigen transfers to induce isotype switching. Some CD19+ B cells co-express CD138 (plasma cells). After seven weeks of stroke in mice, an increase in the level of IgG (IgG1 and IgG2b), IgA and IgM was observed in the lesion area. B cell-deficient μMT −/− mice showed no IgG in the ischemic area and no cognitive deficit after stroke, although infarct size and T cell infiltration were similar to those in wild-type mice. Therefore, it is reasonable to think that cognitive ability after stroke may depend on the antibodies produced by B cells. When mice were treated with CD20 antibodies to ablate B cells, cognitive deficits and IgG expression after stroke were also prevented [111]. 

However, Schuhmann et al. demonstrated that pharmacological B cell depletion using anti-CD20 24 h before MCAO in mice or using B cell-deficient JHD −/− mice that have no circulating B cells did not affect stroke lesion size, neutrophil number, monocytes and TNFα and IL-1β levels at days 1 and 3 post-MCAO [112].

#### 3.3.3. Neuroimmune Crosstalk in the Pathogenesis of Ischemic Stroke

A tight connection exists between the central nervous and immune systems through complex communication networks. The immune system monitors the brain functioning and reacts when cerebral homeostasis is altered because of injuries or diseases. Stroke promotes strong phlogosis, involving the production of cytokines (i.e., TNF-α) by various cytotypes in the brain, including human neurons and activated glial and endothelial cells, with consequent blood–brain barrier detriment and an infiltration of numerous types of leukocytes after a determined gap of time. These cytotypes can play a neuro-damaging or neuroprotective role, and the severity of the cerebral damage is strictly correlated with the balance between these two possible parts. 

The relations between the various cytotypes of the immune system during the acute phase of ischemic stroke is an elaborate mechanism controlled by several factors. The ischemic stroke is a complex pathology, and essential factors, such as the ischemic lesion’s harshness and the stroke’s location, age and comorbidities, can affect the interaction and the equilibrium between the cytotypes in the necrotic cerebral parenchyma. All these factors can influence the local cytokine secretion, critical in modulating the interactions between the immune cells. For example, severe ischemic damage and a raised proinflammatory milieu with massive IL-6 and TNF-α releases may cause an increased neutrophil recall in necrotic cerebral tissue. 

Neutrophils are considered damaging since compelling evidence correlates this cytotype with blood–brain barrier breakdown and brain injury. An increased blood neutrophil count is also associated with larger infarct areas in subjects affected by cerebral ischemia [97]. Furthermore, the role of microglia and macrophages/monocytes during cerebral ischemia depends on the M1/M2 polarization status. Specific cytokines in the local milieu (i.e., M1: IFN-γ, M2: IL-10 and TGF-β) influence the polarization of one of the two phenotypes. 

The prevalence of the M1 phenotype is correlated with more severe ischemic damage, hypoxia-inducible factor-1 (HIF-1) activation and increased anaerobic glycolysis. The polarization of the microglia to the M1 phenotype and the resulting increased production of IL-23 facilitates the recruitment and stimulation of γδ T cells, a subset of unconventional innate T cells with a diverse T cell receptor that could play a detrimental function during acute ischemic stroke. Rising evidence reinforces that γδ T cells are pathogenic in testing cerebral ischemia/reperfusion models by secreting IL-17 and stimulating phlogosis [113].

On the other hand, the prevalence of a local anti-inflammatory milieu stimulated by the secretion of IL-10 and TGF-β facilitates the polarization of the microglia to the anti-inflammatory M2 phenotype, which has a neuroprotective function. Furthermore, releasing these anti-inflammatory cytokines promotes the recruitment of regulatory lymphocytes that play immunomodulatory and immunosuppressor functions in the injured cerebral parenchyma. Indeed, consistent evidence supports the beneficial functions of Tregs in an experimental cerebral ischemia model [97]. While partially understood, the puzzle of the immune system’s interaction during ischemic stroke remains a complex and intriguing study area. The known role of the cytokine environment is just the tip of the iceberg, as these interactions involve intercellular crosstalks by mechanisms yet to be fully comprehended. This complexity underscores the need for further research and piques our curiosity, driving us to delve deeper into understanding the immune response to stroke. The equilibrium between neuroprotective and neurodegenerative actions, a crucial aspect of this response, is influenced by several factors, reflecting the heterogeneous nature of ischemic stroke.

## 4. Hemorrhagic Stroke Pathophysiology

Hemorrhagic stroke happens less frequently than an ischemic stroke but has a high morbidity and mortality rate (about 40%) [114]. Hemorrhagic stroke primarily occurs due to blood vessel rupture and the extravasation of blood in either the brain parenchyma and ventricles, called intracerebral hemorrhage (ICH), or the subarachnoid space, called subarachnoid hemorrhage (SAH). ICH can also happen after ischemic stroke, and is associated with hematoma expansion, edema and intraventricular hemorrhage. The leading causes of bleeding include hypertension, the use of anticoagulants and thrombolytic agents and head injury [115]. Other causes of hemorrhagic stroke and neurologic impairment and disability include cerebral aneurysms. Described as the ballooning or pouching of the vessel wall, a fully developed and untreated aneurysm continues to grow and expand until it ruptures, leading to a hemorrhage.

### 4.1. Brain Injuries after Intracerebral Hemorrhage

Bleeding following ICH causes a disruption of the brain structure within hours, and it is almost impossible to prevent damage due to the primary lesion [116]. During the first day of intracranial hemorrhage, the critical factors determining the post-ICH clinical outcome are the expansion of the hematoma and the increase in hemorrhagic volume [117]. Secondary injury results from intraparenchymal hematoma, resulting in multiple injurious events and neurological deficits [118]. Various blood components activate cytotoxic, excitotoxic, oxidative and inflammatory pathways [119]. Thrombin, iron and hemoglobin from the hematoma are the major contributors to secondary brain damage post-ICH [120].

### 4.2. Oxidative Stress and Hemorrhagic Stroke

Oxidative stress plays a very important role in the brain injury that can develop after an intracranial hemorrhage. Oxidative stress is described as the lack of balance between the production and the ability to eliminate ROS by the physiological antioxidant mechanism of the cells [121,122].

Superoxide radicals (O_2_−), hydrogen peroxide (H_2_O_2_) and hydroxyl radicals (OH) constitute the primary forms of ROS [122]. Oxidative stress contributes to the growth and progression of perihematomal edema in brain hemorrhage patients [123]. ROS damage the central nervous system through cell death and structural damage, especially blood–brain barrier disruption. Apoptosis releases excess free radicals, which induces lipid and nucleic acid peroxidation through various pathways [124]. Furthermore, H_2_O_2_ can alter mitochondrial function and upregulate the expression of pro-apoptotic genes, ultimately inducing apoptosis following ICH [125].

### 4.3. Neuroinflammation in Hemorrhagic Stroke

As already mentioned above, intraparenchymal blood activates various inflammatory pathways. Recently, it has been shown that these immune pathways are very similar in ischemic and hemorrhagic stroke [99]. Hemorrhagic stroke can initiate inflammatory responses, induce cerebral edema and disrupt the BBB with neurotoxic materials such as thrombin, fibrin and erythrocytes [126]. The accumulation of erythrocytes can initiate inflammation by activating toll-like receptor 4 (TLR4) in the microglia to release TNF α and IL-1β [127]. Thus, local microglia and astrocytes are the first cells to respond to the ICH.

Their role is to promote, once activated, the influx of circulating macrophages. Subsequently, various pro-inflammatory cytokines are expressed, further activating lymphocytes and ultimately limiting ICH-induced damage by disrupting the BBB [128]. The NLR family, a pyrin domain containing three inflammasomes (NLRP3), induces inflammation via caspase-1 and interleukin (IL)-1β post-ICH [97]. In the same way, TLR4 activates an inflammatory pathway of ICH and neuronal apoptosis [129]. Damage-associated molecular patterns (DAMPs), such as that of HMGB1, a non-histone nuclear protein with a pro-inflammatory action, are other key players in the inflammatory processes of ICH and are essential when released into the extracellular space [124].

The secreted HMGB1 interacts with TLR-2 and TLR-4 to trigger inflammation. HMGB1 secreted by monocytes and macrophages was observed to interact with TLR2, TLR4 and TLR5 to upregulate the nuclear factor kappa-light-chain-enhancer of activated B cells (NF-kB). 

Studies involving animal models of ICH have shown that HMGB1 levels are increased in peri-hematoma regions post-ICH [130]. Furthermore, stroke severity is significantly correlated with increased serum HMGB1 levels [131].

Thus, the expression level of HMGB1 in plasma and CSF can be related to SAH outcomes. As the inflammation proceeds, leukocytes are recruited via adhesion molecules to secrete diverse chemokines and cytokines to induce endothelial cell death, recruit more immune cells and damage the tight junctions in the BBB [126]. Microglia and macrophages phagocytose erythrocytes and degrade debris after the injury [132]. T cells can be detrimental, but Tregs can be neuroprotective in rat models of hemorrhagic stroke, as mentioned for ischemic stroke [133].

## 5. New Epigenetic Players in Stroke Pathogenesis: From Non-Coding RNAs to Exosomal Non-Coding RNAs

MiRNAs are essential in modulating gene expression in all types of cells. Binding to messenger RNA (mRNA) can cause cleavage or inhibit translation into proteins. The process of miRNA biosynthesis is a complex biological process which involves the transcription of primary miRNA (pri-miRNA) followed by processing with the nuclear endonuclease Drosha to create pre-miRNA. Once transported to the cytoplasm, the enzyme Dicer processes the pre-miRNA to form a short double-stranded RNA sequence. One of the chains is degraded, leaving behind the mature miRNA. 

The mature miRNA then binds to Ago2, forming RNA-induced gene silencing complexes (RISCs) that enable it to interact with mRNA targets to cause degradation or translational repression. Notably, a single miRNA can regulate the expression of hundreds of mRNAs, and, vice versa, an mRNA can present various sequences to interact with multiple miRNAs [134].

An alternative biosynthetic pathway for miRNAs exists, where some miRNAs are produced from specific introns called “mirtrons.” Mirtrons are of the size required to synthesize pre-miRNAs, eliminating the need for Drosha endonuclease directly. This pathway adds another layer of complexity to the miRNA biogenesis process. After pre-miRNAs are synthesized, they are transported to the cytoplasm, where they undergo processing by the Dicer enzyme. The Dicer enzyme cuts the pre-miRNAs to produce short double-stranded RNA sequences that are 19–25 nucleotides long. These double-stranded RNA molecules are then loaded into the RNA-induced silencing complex (RISC), which leads the RISC to the target messenger RNAs (mRNAs), influencing the mRNA translation. 

To conclude, the biosynthetic pathway of miRNAs is a complex multi-step mechanism involving multiple steps and pathways. The alternative pathway through mirtrons adds another layer of complexity to the process and highlights the diversity of miRNA biogenesis [135].

### 5.1. Mechanism of Action

Typically, the miRNA binds to a complementary sequence in the 3’UTR region of the target mRNA. However, new findings indicate that binding can occur in the 5’UTR region. The “seed region,” spanning nucleotides 2 to 8 of a mature miRNA, is crucial for recognizing the target mRNA [136]. 

It is crucial to note that a miRNA can regulate the expression of multiple mRNAs. Similarly, an mRNA can have several sequences interacting with different miRNAs simultaneously. Additionally, it is essential to consider that miRNAs are not immediately degraded after interacting with a single target. Therefore, they can demodulate other mRNAs as well [137].

Identifying miRNA target genes is a complex process that requires careful consideration. Several elements can affect the accuracy of the identification process, such as the accessibility of the target sequence and the secondary structure. Despite these challenges, researchers continue to work towards identifying miRNA targets with greater precision than those of mRNAs [138].

The role of different miRNAs in ischemic stroke’s pathogenesis is summarized in Table 2.

### 5.2. Circulating miRNA as a Biomarker

Given the current situation, it is critical to find an examination that is both highly sensitive and effective while also being budget-friendly and non-intrusive to identify and predict the prognosis of hemorrhagic or ischemic stroke and its subtypes. Biomarkers can shed light on these diseases’ underlying mechanisms. They may be proteins, nucleic acids, or metabolites and can provide valuable information on a given disease’s risk, diagnosis, severity and prognosis through their quantification [155].

Physicians use brain imaging to determine the most appropriate treatment for stroke patients. However, this method has certain limitations, such as high costs, contraindications and the need for expert radiologists to interpret the results. Additionally, access to brain imaging services is often limited. As a result, researchers are working hard to develop more reliable tools to help healthcare providers manage stroke patients effectively. Unlike other common diseases like diabetes or acute myocardial infarction, there is no specific or sensitive biomarker available to aid in the diagnosis and treatment of stroke. This fact is mainly due to the complexity and heterogeneity of the disease, as well as the impact of the blood–brain barrier on the circulation of biomarkers [156].

### 5.3. Exosomes Biogenesis

Extracellular vesicles (EVs) are a new promising study area. These small or large lipid bilayer membrane particles are released from all living cells into the extracellular environment. Exosomes, the smallest type of EVs, have an average diameter ranging between 30 and 150 nm and have been the most studied. It has been discovered that EVs play an active role in different biological mechanisms, including intracellular homeostasis and intercellular communication. These vesicles may hold the key to finding a reliable biomarker for stroke diagnosis and treatment [157,158].

Exosomes are derived from endosomal structures that originate through the endocytosis of invaginated endosomes from the plasma membrane [159].

Those tiny vesicles are released from cells and carry necessary molecular signals to other cells, which can affect the recipient cells in various ways. These vesicles can be loaded with specific cargo, and their release can be regulated by environmental and cellular cues such as stress, inflammation or cell-cycle events. Exosomes travel through the circulation system to reach their target cells, where they can bind to the cell surfaces and get absorbed through specific mechanisms. Recent studies suggest that the uptake of exosomes can be regulated and that certain classes of exosomes may contain specific targeting molecules that lead to some degree of specificity towards specific recipient tissues [160,161].

Exosomes can also undergo transcytosis, allowing them to cross the blood–brain barrier, gaining access to the central nervous system (CNS) [162].

### 5.4. Exosomes in Brain Injury

Cells release exosomes into biofluids such as cerebrospinal fluid (CSF). These vesicles are enriched with tetra-spanin proteins (CD81 and CD63), Alix (a regulator of the endosomal trafficking pathway) and chaperone protein HSP70. However, the volume of exosomes varies according to the cell origin and the pathological and physiological conditions. 

Various studies have shown that exosomes carry cargoes of proteins, RNAs and lipids such as miRNAs and mRNAs. Through centrifugation and other procedures, it is possible to isolate exosomes from biofluids and the supernatant of cells that have been cultured in an exosome-free medium. Proteomic and RNA analyses have shown that exosomes are a rich source of biomolecules with potential applications in diagnostics and therapeutics [163,164].

Various cell types can release exosomes in the CNS during brain injury and may play a significant role in post-stroke brain remodeling [165].

The information of neural death in the ischemic brain is communicated through exosomes to other cells in the brain. Because of this, miRNAs in exosomes play a crucial role in intercellular communication [166].

Astrocytes release exosomes under both physiological and pathological conditions. These exosomes contain various biological molecules such as DNA, miRNA and proteins, but their composition varies depending on the stimuli. Under normal conditions, astrocyte-derived exosomes contain neuroprotective and neurotrophic elements and molecules that help in neurite outgrowth, synaptic transmission and neuronal survival. Numerous studies suggest that astrocytes are activated during cerebral ischemia and secrete exosomes to protect the central nervous system. Pei et al. demonstrated that astrocyte-derived exosomes inhibit autophagy and enhance neuronal viability in an ischemic stroke model [167].

Moreover, astrocyte-derived exosomes also contain miRNAs, such as miR-34c and miR-361, that protect neurons and prevent nerve damage after cerebral ischemia [168,169]. 

The detection of stroke biomarkers in the bloodstream is challenging because of the blood–brain barrier (BBB), which acts as an interface between the central nervous system and the peripheral circulation. As a result, biomarkers from cerebrospinal fluid (CSF) have difficulty transitioning into the bloodstream. However, exosomes have shown potential to cross the BBB, making them a promising candidate for being measured in the blood. Some authors have recently explored the use of exosomes as stroke biomarkers. Patients who have had an ischemic stroke show different cargo exosomes than those of healthy controls. miRNAs, the most investigated exosome component, could provide valuable information.

### 5.5. miRNA as a Biomarker in Clinical Practice

Being able to quickly diagnose stroke represents an important medical need, since stroke diagnosis takes a long time, mainly because various neuroimaging techniques are used, and it is also dependent on the healthcare provider. The functions of miRNAs were first found in original tissue samples. Most studies have focused on correlating miRNAs present in brain tissues with the pathophysiological mechanisms underlying stroke, such as cellular apoptosis, inflammation and oxidative stress [139,140,170,171].

Tan et al. conducted a study comparing miRNAs’ expression in healthy individuals and those diagnosed with cerebral ischemia. They used microarray analyses of blood specimens and selective TaqMan quantitative polymerase chain reactions (qPCRs) of miRNAs to evaluate miRNA expression. The study found that miR-25, miR-125b-2, miR-125b-627, miR-125b-27a, miR-125b-488 and miR-145 are significant biomarkers for the diagnosis and treatment of stroke. These miRNAs may be relevant in developing effective treatments for cerebral ischemia [141].

In their study, Wang et al. analyzed miRNAs circulating in the plasma of patients diagnosed with acute stroke and pathologies other than stroke. The goal was to determine whether such miRNAs could be used as biomarkers for both the diagnosis and treatment of stroke [172]. 

Several miRNAs have been identified as potential biomarkers for diagnosing certain conditions. These include miR-106b-5p, miR-126, miR-30a, miR-4306, let-7b27 and let-7b28. Studies have correlated high serum miR-130a levels with severe brain edema and poor prognosis following acute intraparenchymal hemorrhage (ICH) [173]. 

Wang et al. compared the expression of circulating microRNAs in blood and hematoma samples and found that the levels of 59 miRNAs significantly decreased after ICH, and Hsa-miR-21-5p was reduced in both peripheral blood and hematoma samples after ICH [142,174].

### 5.6. miRNA as a Biomarker in Acute Ischemic Stroke

Among the various miRNAs considered, miR-101a-3p was considered as the most important ischemic stroke biomarker for further future evaluation. miR-101a-3p expression levels increase at early time points, 30 min and 180 min, after the induction of permanent ischemia, and at a longer time point (approximately nine hours) after transient ischemia. However, no changes were observed following hemorrhagic stroke [175].

According to research by Liu et al., miRNAs can play both beneficial and harmful roles. Their research discovered that suppressing the expression of miR-155 can enhance the proliferation, migration and tube formation ability of human brain microvessel endothelial cells by reducing cellular apoptosis and (ROS) production [176].

Researchers found that inhibiting miR-27b could alleviate neurological deficits by suppressing neuroinflammation and reducing cell death. Additionally, miR-155 and miR-124 were reported to play a role in macrophage polarization [177].

miR-134 can facilitate the remodeling of neuronal structures by inhibiting the translation of Limk1-mRNA, a protein kinase that affects dendritic spine development [178].

Some miRNA types can exert multiple functions, such as miR-181a, which can improve cellular survival by suppressing inflammation responses in monocytes and macrophages [179].

Moon’s report demonstrated that inhibiting miR-181a reduced neuronal apoptosis induced by forebrain ischemia [143].

### 5.7. miRNA in Hemorrhagic Stroke

Studies have shown that specific miRNAs (microRNAs) can be used as biomarkers to differentiate between subarachnoid hemorrhage (SAH) patients with delayed or non-delayed cerebral infarction and controls. Similarly, a specific panel of miRNAs in cerebrospinal fluid can distinguish SAH patients from controls and differentiate between SAH patients with vasospasm and those without it. In addition, clinical studies have identified that serum miR-130a or a panel of blood-specific miRNAs can differentiate between intracerebral hemorrhage (ICH) patients and controls. Moreover, plasma miR-29c and miR-122 can help distinguish between the hematoma and non-hematoma enlargement groups of ICH patients [170]

The miRNA levels can be increased or decreased to treat or prevent hemorrhagic stroke. In an experimental model of ICH, injecting a miR-130a inhibitor into the right lateral ventricle before ICH induction in male rats reduced the expression of miR-130a, decreased brain edema and improved neurological function [174].

A miR-367 mimic can increase miR-367 levels, inhibit the inflammatory response and decrease brain edema and neurological injury in mice with ICH [144].

The overexpression of miR-223 through a miR-223 mimic injection in ICH mice reduces brain edema and improves neurological functions while inhibiting the inflammatory response [145].

### 5.8. miRNA as Target Therapy: The of Role miRNA Mimics

MiRNA-based therapeutics hold significant potential in combating various diseases and can be broadly categorized into two types—miRNA mimics and miRNA inhibitors. 

MiRNA mimics can help compensate for the loss of specific miRNAs that are downregulated and correlate with disease progression. In contrast, miRNA inhibitors (or antimiRs) can suppress the overexpressed miRNAs that contribute to disease pathogenesis. To imitate miRNA precursors, miRNA mimics are designed as synthetic short double-stranded oligonucleotides. Once introduced into cells, these oligonucleotides can be recognized and processed using miRNA biogenesis machinery. To ensure their effectiveness, miRNA mimics are constructed with one “guide strand” and one fully or partially complementary “passenger strand”, which plays a crucial role in regulating gene expression [180,181]. 

### 5.9. miRNA as Target Therapy: The Role of Anti-miR

Numerous studies have proved the therapeutic benefits of the intravenous infusion of antagomiR in cardiovascular pathologies. A promising new technique called “miRNA masking” has been developed to improve the specificity of AMO (anti-MIRNA-oligonucleotides). This technique selectively inhibits the binding of a particular miRNA to a target mRNA without interfering with other targets [182]. 

Although AMOs have been found to be dose-dependent and require high doses for effective inhibition, a new strategy called “miRNA sponges” promises to overcome this limitation. These RNA sequences present multiple binding sites for miRNAs and can be designed to interact with all members of a miRNA cluster, achieving the inhibition of an entire functional class. These observations provide hope for a possible use of these molecules in treating hypercholesterolemia [183,184,185]. 

### 5.10. The Role of Angiogenesis as a Potential Target

Angiogenesis is a natural process in which new blood vessels are formed from existing ones. While the growth of blood vessels is tightly regulated in adult brains under normal conditions, research from both human and animal stroke models indicates that neovascularization occurs in the adult brain after cerebral ischemia [186,187].

Studies have shown that the development of new blood vessels, known as post-ischemic angiogenesis, is critical in restoring blood flow and neuronal metabolism following a stroke. This cumulative evidence highlights the importance of angiogenesis as a potential therapeutic target in stroke recovery [188].

For instance, when a miR-107 mimic was used, it lessened the ischemic brain infarction and increased the number of capillaries in the penumbral area, possibly boosting the endothelial VEGF165/164 levels, which promoted angiogenesis [146].

Post-stroke angiogenesis contributes to improved neurological recovery by promoting tissue repair, vascular remodeling and plasticity in both stroke patients and animal stroke models (cit1 e cit2) [189,190].

Other findings by Sun P. et al. show that several miRNAs play essential roles in regulating post-stroke angiogenesis, including miR-107 and miR-150. They demonstrated that deleting the miR-15a/16-1 cluster in the endothelium had a potent pro-angiogenic effect following reperfusion of the ischemic brain. This fact led to significant improvements in long-term neurological recovery after ischemic stroke. This study confirmed that vascular remodeling occurs during the prolonged recovery phase and showed that the endothelium-targeted removal of the miR-15a/16-1 cluster improved revascularization and angiogenesis in peri-infarct brain areas thereafter in ischemic stroke patients. Therefore, endothelial miR-15a/16-1 could be a promising pharmacological target to improve post-stroke neurological recovery by enhancing cerebral angiogenesis [147].

### 5.11. The Role of Synaptic Plasticity as a Possible Target

The results of a recent study by Xin, Wang and their colleagues have uncovered an exciting new development in stroke research. Their research shows that miR-17-92-enriched exosomes can promote neural plasticity and improve recovery in rats post-transient middle cerebral artery occlusion. The downstream effector proteins in these exosomes can promote neurite remodeling, axonal growth and cell proliferation in primary cortical neurons, leading to enhanced functional recovery. Moreover, when miR-133b enriched exosomes were administered to a stroke model, further exosomes were released from glial cells and astrocytes, providing trophic support to axons and ultimately leading to increased neuronal plasticity and stress protection. The study highlights the significant therapeutic benefits of neuroprotective miRNA-enriched exosomes, which can improve neuroprotection and recovery under oxidative stress conditions. These findings are truly remarkable and offer hope for future stroke treatments [148,191]. 

### 5.12. The Role of Post-Stroke Inflammation

In a groundbreaking study by Xu et al. [149] a promising mechanism to combat post-stroke inflammation was discovered. The researchers identified the suppression of toll-like receptor 4 (TLR4) by miR-1906, which triggers the proinflammatory cascade in the brain after a stroke. By administering miRNA-1906 agomir to rats, the team found that TLR4 protein levels were significantly reduced, despite the presence of TLR4 mRNA, indicating that miRNA-1906 suppresses TLR4 translation rather than TLR4 mRNA degradation. Consequently, the downstream signaling pathways that activate proinflammatory genes are inhibited, reducing post-stroke inflammation, infarct volume and peri-infarct tissue damage. Xu et al.’s findings offer a promising avenue for developing targeted therapies for post-stroke inflammation, which could improve patient outcomes and quality of life [149].

### 5.13. miRNA Involved in Neuroprotection

The highly expressed miR-204-5p (2075 times), miR-125b-5p (108 times), miR-9-5p (299 times), miR-338-3p (146 times), miR-187-3p (21 times) and miR-9-3p (42 times) in cerebrospinal fluid were associated with the regulation of matrix metalloproteinase-9 (MMP-9), interleukin (IL)-1b, IL-6, occludin and selectin E. The miRNA profiles in cerebrospinal fluid were physiologically close to those in brain extracellular fluid. However, only a small number of studies have been reviewed. It would, therefore, be appropriate to focus further studies on the function of miRNAs in the cerebrospinal fluid of stroke patients [150].

miRNA 21, miR-99a and miR-497 have been found to reduce ischemic volume and protect neuronal cells from apoptosis, thus improving neurological functions in rats and in vitro models of ischemic stroke [151,152,153,192,193,194,195,196,197,198,199]. 

Except for the functions mentioned above, the overexpression of miR-424 and miR-let-7c-5p could also suppress the activation of microglia in cerebral ischemia [153].

The excessive secretion of miR-126, miR-132, miR-103 and miR-367 can reduce neurobehavioral and neuropathological changes in hemorrhagic stroke by improving BBB integrity and reducing neuroinflammation and neuronal apoptosis [144,192,193,194]. 

Finally, an excess of miR-210 could help angiogenesis and neurogenesis in the mouse brain and improve the repair of damaged brain tissues [195].

### 5.14. Future Perspectives

In the future, pre-miRNAs and anti-sense RNAs will be synthesized and administered intravenously to target the abnormal DNA translational control, which can either increase or decrease miRNA levels. However, extensive research is needed to establish the specificity and sensitivity of such treatments and potential side effects and uptake/elimination mechanisms. Additionally, using engineered exosomes and microvesicles that can deliver therapeutic miRNAs to the site of brain injury could open up new avenues for stroke therapy. Although some studies have shown promising results, proof-of-concept studies are still required to determine whether such therapies can improve stroke outcomes.

## 6. Molecular Mechanisms and Therapies in Stroke: Update on Recent Developments

In recent years, researchers aimed to gain a better comprehension of the molecular mechanisms underlying ischemic stroke and the consequent cerebral damage. Understanding different pathways could lead to developing new therapies, apart from the classic thrombolysis, improving the chances of a better neurological outcome.

### 6.1. Inflammation

At the beginning of the ischemic process, inflammation contributes to the disruption of the blood–brain barrier (BBB), causing edema; on the other hand, at a later time, it contributes to fixing the damaged cerebral tissue [196]. Following an ischemic stroke, microglia become M1- or M2-activated macrophages. M2 ones have an anti-inflammatory function, promoting production factors such as insulin-like growth factor 1 (IGF-1) and brain-derived neurotrophic factor (BDNF). M1 macrophages have a crucial function in the release of multiple factors, either involved in the development of further inflammation or toxic for the neuronal cells, such as NO, ROS, IL-6, TNF-α and IL-1B [197]. IL-1, especially IL-1β, promotes a further production of IL-6 and TNF-α, triggering the other cytotypes involved in inflammation; this entire process leads to further injury to the cerebral tissue, compromising the neurological outcome [198].

In addition, inflammation promotes the overexpression of adhesion molecules, such as E-selectin and ICAM-1, that let dendritic cells, astrocytes and lymphocytes reach the cerebral tissue affected by ischemia [199]; furthermore, this amount of cells interferes with blood circulation [200]. During phlogosis, it is possible to observe an increased production of matrix-metalloproteases (MMPs) capable of harming the BBB, with consequent edema and cerebral injury; their expression is, in fact, correlated with the entity of cerebral damage and the successive chance of bleeding [201]. Clausen et al. observed that etanercept, a monoclonal antibody against TNF-α, improved the neurological outcome in mice affected by induced ischemic stroke, but not the extent of the infarcted area yet [202]. 

cTfRMAb-TNFR, capable of moving TNFR crosswise across the BBB, was able to improve the neurological injury and decrease the size of the ischemic cerebral portion [203].

Both sTNF-αR1 and solTNFR1 have been tested in animal models, revealing a significant reduction in inflammation and improved axonal remodeling [204]; conversely, they could increase the chance of demyelination and atherosclerosis [205]. Various research works demonstrated that IL-1Ra decreased the damage caused by ischemic stroke in animal models [206]; in addition, recombinant human interleukin-1 receptor antagonist (rhIL-1Ra) seemed to have similar properties in a randomized, double-blinded, placebo-controlled trial [207]. 

### 6.2. Excitotoxicity

Ischemia leads to a lack of oxygen, causing an increased production of glutamate and a consequent activation of Na^+^/Ca^2+^ channels associated with N-methyl-D-aspartate receptors (NMDARs) [208], provoking an excessive burden of Ca^2+^ in cytosol and mitochondria [209]. This process is called excitotoxicity and, by kainite receptors, NMDARs and α-amino-3-hydroxy-5-methyl-4-isoxazolepropionic acid [210], promotes the release of free radicals and the activation of lipases, kinases and proteases [211], resulting in apoptosis [212]. On the other hand, it seems that particular NMDARs in the synaptic space containing the GluN2A subunit have a protective role in reducing oxidative stress and, consequently, excitotoxicity [213].

The Tat-NR2B9c peptide is able to modulate the NMDARs with a potential therapeutic role in ischemic stroke. In fact, multiple studies in animal models showed that this molecule decreased the ischemic cerebral area with a better neurological outcome [214,215,216]; ZL006 and IC87201 are two little molecules capable of modulating NMDARs and showed similar results in ischemic stroke [217,218]. Hong et al. studied the possible use of Neu2000, an NMDAR antagonist, demonstrating a beneficial role in ischemic stroke [219]. Unfortunately, all these molecules which antagonize the NMDARs’ pathway do not have a clinical application because of the normal functions of these receptors and, consequently, all the side effects, such as cardiovascular and neuropsychiatric diseases [220].

### 6.3. BBB Alterations and Matrix Metalloproteases (MMPs)

In animal and human stroke models, an increased release of matrix metalloproteases (MMPs) was observed [220], promoting BBB disruption, cerebral edema formation and further inflammation [221,222]. Barr et al. reported that MMP9 has a crucial role in damaging the BBB, increasing its permeability [223]; similarly, Chelluboina et al. observed increased levels of MMP12, and its silencing was correlated with a better neurological prognosis [224]. The role of MMP2 in ischemic stroke is still unclear, but it seems to interact with VEGF to repair the BBB [225].

Since their crucial role in BBB disruption, MMPs’ repression may have a role in ischemic stroke therapy. Hydrogen sulfide and the triggering of the vagus nerve seem capable of inhibiting MMP9, protecting the BBB [226,227]; in addition, even the administration of norcantharidin suppressed this metalloproteinase, protecting the BBB [228]. Michalski et al. observed a correlation between high-pressure oxygen and BBB integrity, possibly modulating MMP2 [229].

### 6.4. Inflammasomes

Inflammasomes are formed by a group of proteins [230] and take part in inflammation during ischemic stroke, causing apoptosis [231]. NLRP3 inflammasomes seem to participate in cerebral inflammation, modulating microglia [232] by the NF-κB pathway [233]; on this basis, NLRP3 inflammasomes may represent a therapeutic target in cerebral ischemia, decreasing inflammation [234]. Wang et al. reported that genistein, a phytoestrogen, can restrain the formation of this inflammasome, reducing the neurological deficit in mice models [235]; similarly, MCC950, capable of blocking NLRP3, reduced caspase-3-mediated cell death and decreased the cerebral ischemic areas [236].

### 6.5. Chemokines

Chemokines are little proteins with signaling functions crucial during neuroinflammation in cerebral ischemia. These proteins, such as microglial response factor-1, cytokine-induced neutrophil chemoattractant and fractalkine, are stimulated by all the proinflammatory cytokines [237]. During ischemic stroke, chemokine ligand 2 (CCL2) and its receptor, CCR2, are overexpressed and promote the infiltration of immune cells in the ischemic area, leading to further inflammation [238,239,240]. Takami et al., reported a detrimental effect of the administration of CCL3 during ischemic stroke in animal models [241].

In animal models, silencing the CCL2 or CCR2 genes resulted in a smaller ischemic area and a better neurological outcome [240,242]; on the other hand, silencing these genes reduced the release of anti-inflammatory cytokines [243]. 

### 6.6. Hypoxia-Inducible Factor (HIF) 

HIF is a protein produced during ischemia able to stimulate transcription and is formed by HIF-1α and HIF-1β [244]; HIF-1 promotes the switch to anaerobic cellular metabolism, supporting cells during hypoxia [245]. Ziello et al. reported that ROS stimulate the release of HIF-1α [246]; consequently, HIF-1 needs an oxidative environment to work. On the contrary, several studies showed that HIF-1α could preserve brain cells during ischemia: for example, Kim et al. observed that decreasing HIF-1α in animal ischemic models resulted in worse neurological dysfunction [247]; in addition, HIF-1α seems to preserve the neuron’s integrity during oxidative stress [248].

### 6.7. Cell-Based Therapies

Stem cells seem a promising therapeutic option since they participate in neuroplasticity [249]. Neural stem cells (NSCs) can turn into different cerebral cytotypes, such as neurons, glial cells, astrocytes and oligodendrocytes [250] and can develop new cells over time [251]; on this basis, Baker et al. reported an improved neurological outcome in animal ischemic stroke models after NSC therapy [252]. Furthermore, Zhang et al. reported the possibility of combination therapy with NSC, brain-derived neurotrophic factors and vascular endothelial growth factors [253]. 

Hematopoietic stem cells (HSCs) have been proposed as a possible treatment for ischemic stroke [254], collected using peripheral blood and successively administered intravenously [249]; in fact, Modi et al. observed that HSC therapy resulted in a better prognosis in animal models [255].

Human umbilical cord blood-derived mesenchymal stem cells (HUCB-MSCs) seem to be effective in multiple pathological animal models [256]. Unlike embryonic cells, this cytotype is unaffected by immune reactions, does not develop tumors and is not restricted by any ethical controversies [257].

### 6.8. Drug-Based Therapies

At the moment, tissue Plasminogen Activator (tPA) is the only therapy approved for ischemic stroke, but it has a short therapy window, several contraindications and does not play a role in neuroplasticity [258]; due to this, only a tiny percentage of patients can benefit from this treatment.

Fibrinogen-depleting agents derived from viper venom and could represent a possible alternative to tPA [259]; these molecules can improve blood perfusion in ischemic areas by clearing blood clots [260].

Gamma-aminobutyric receptor (GABA) agonists, such as diazepam and midazolam, seem to reduce neurological symptoms and protect brain cells from inflammation during ischemia [261].

The inhibition of calcium and sodium channels could find a role in the treatment of ischemic stroke; amlodipine, a Ca2+ channel antagonist, decreased the chance of ischemic stroke in patients affected by essential hypertension by 13.5% [262]; in addition, Frank et al. reported that blocking sodium channels interferes with cellular depolarization, making brain cells less excitable during ischemic stroke [259].

As said above, excitotoxicity is mainly mediated by glutamate receptors and has a detrimental effect on cerebral cells [263]; since these receptors are ubiquitous, researchers focus on blocking the NMDAR pathways rather than the receptors, causing fewer side effects [264].

DM199 is a synthetic form of human tissue kallikrein-1 (KLK) that is able to improve blood circulation and promote the development of new vessels without the contraindications and side effects of tPA or thrombectomy; in China, this type of treatment is already used for acute cerebral ischemia (Kailikang^®^) after several studies and a large trial were conducted.

To conclude, cofilin inhibitors could be a potential treatment in ICH; cofilin is a protein with a crucial role in cytoskeleton formation and has a pathological function in different neurological pathologies [265], promoting inflammation and cellular injury. Alaqel et al. showed that inhibiting cofilin in an ICH animal model improved the neurological outcome [266].

## 7. Conclusions

It is now known that stroke’s consequences are not limited to the central nervous system but are instead characterized by the influx and efflux of cytokines, cells and fluids, which can significantly modify the stroke’s outcomes.

Neuroinflammation, oxidative stress and excitotoxicity are among the protagonists of the interactions within this complex mechanism. Another topic of great interest concerns the use of non-coding RNAs (ncRNAs), which play an important role in angiogenesis and recovery from ischemic cerebral lesions, thus proving essential in the field of neuroprotection. In fact, recent evidence suggests the potential role of microRNAs and long non-coding RNAs (lncRNAs) as potential useful elements in the diagnosis, therapy and prognosis of brain pathologies, including ischemic and hemorrhagic stroke. A crucial role also appears to be played by exosomes, nanocarriers that play important intercellular mediating roles in neuroreparative events that follow neuronal damage. It is, therefore, clear how the detailed understanding of these molecules, which are the basis of the pathogenesis of stroke, is necessary for new therapeutic perspectives, which are crucial considering that classic treatments such as tPA only have a limited therapeutic window. Given tPA’s limitations, another promising and much more versatile therapy for patients appears to be DM199, which significantly increases the rate of recovery in affected individuals. Other targets for treatment have also been identified, such as glutamate receptors, GABA receptors, sodium and calcium channel blockers and fibrinogen-depleting agents, which, when altered or targeted, reduce symptoms and improve functional outcomes. 

Stem cell research mainly focuses on post-stroke recovery, including neural stem cells, HSC stem cells and HUBC-MSC stem cells. Overall, stroke therapeutic research is on track to develop safe and efficient therapies for stroke. Much work has been carried out in stroke research to find potential molecular targets and generate new avenues for researchers.

## Figures and Tables

**Table 1:** A table summarizing the temporal trends, the actions of each immune cell after stroke and the produced cytokines and chemokines. IL: interleukin; TNF: tumor necrosis factor; TGF: transforming growth factor; BBB: blood–brain barrier; MMP: matrix metalloproteinase; VEGF: vascular endothelial growth factor; ROS: reactive oxygen species; PDGF: platelet-derived growth factor; DAMP: damage-associated molecular pattern.

Immune Cells	Temporal Trend	Produced Cytokines/Chemokines	Action(s)
Neutrophils	Accumulate after 3 h, peak at day 1–3 and dissipate over 7 days	Elastases MMP-9 IL-1, VEGF ROS, MMP-9 Annexin-1 Resolvins Protectins	Cerebral edema, BBB destruction and neuronal death Degradation of DAMP signaling and vascular remodeling Cerebral angiogenesis Microglia migration toward the infarct core after 1 day Decrease neutrophil migration and pro-inflammatory cytokine release
Mast cells	Significant increase after 24 h	Histamine Heparin Vasoactive agents Chymase MMP-2, 9	Destruct BBB, increase vascular permeability, leukocyte recruitment, cerebral edema, destroy tight junctions and disrupt hemostasis
Monocyte/Macrophage	Shown as early as 3 h, peak at day 3 and become anti-inflammatory at day 7	TNF-α IL-1β IL-10, 23 TGF-β PDGF CD302, 163, 206 Fibronectin 1 Arginase 1	Augment immune responses IL-17a production from T cells Tissue repair and wound healing
4NK cells	3 h, peak at 12 h and remain elevated at least 4 days	IFN-γ IL-17a, 6, 12, 1β TNF-α ROS	Augment immune responses and development of cerebral infarction
CD4^−^/CD8^−^ T cells	1–3 days	TNF-α	Augment immune responses
CD8+ T cells	Detected as early as 3 h and stay for about 30 days	Perforin Fas ligand	Neurotoxicity and augment immune responses
CD4+ T cells (Th1 and Th17) CD4+ T cells (Th2)	Shown at 24 h and stay for about 30 days	IL-2, 12, 17, 21, 22, 23 TNF-α IFN-γ IL-4, 5, 6, 10, 13	Augment immune responses Immunosuppression
Tregs	Shown after several days and stays for about 30 days	IL-10 IL-17 (in certain conditions)	Suppress astrogliosis, regulate astrocyte neurotoxicity and functional recovery Inhibit CD4+ T cell proliferation
B cells	Delayed appearance after weeks of onset	IL-10	Neuroprotection

**Table 2:** This table provides an overview of studies that have analyzed the role of miRNAs in the pathogenesis of ischemic and hemorrhagic stroke. MMP: matrix metalloproteinase; MCAO: middle cerebral artery occlusion; TNF: tumor necrosis factor; NF: nuclear factor; ICH: intracerebral hemorrhage; HIF: hypoxia-inducible factor; VEGF: vascular endothelial growth factor; FGF: fibroblast growth factor; TLR: toll-like receptor; SHIP1: inositol polyphosphate 5-phosphatase 1; SOCS1: suppressor of cytokine signaling 1; SMAD: small mother against decapentaplegic; and TAB: TGF-beta activated kinase 1 binding protein 2.

Authors	Stroke Type	MIRNA Involved	MIRNA Profile	Role
Zhang et al. [139]	Post-ischemic neuronal damage	miRNA-181c	Lower	miRNA-181 suppress TNF-a expression
Wen et al. [140]	Ischemic	miRNA-124	Increased with MCAO (middle cerebral artery occlusion)	miR-155 can exert both pro- and anti-inflammatory effects by targeting different mediators of inflammatory signaling, such as SHIP1, SOCS1, SMAD2 and TAB2
Tan et al. [141]	Ischemic	miRNA 126 miRNA 130	Increased Increased	Endothelial cell/CV functions Angiogenesis
Wang et al. [142]	Hemorrhagic	miRNA-126 miRNA 21-5p	Lower Lower	Endothelial cell/CV functions Protective role against ischemia-induced apoptosis
Moon et al. [143]	Ischemic	miRNA-181	Increased in infarct core; decreased in penumbra after focal ischemia	miR-181 was also shown to sensitize glioblastoma cells to apoptosis by reducing Bcl-2
Yuan et al. [144]	Hemorrhagic	miRNA-367	Lower	miR-367 was a crucial regulator of TLRs downstream NF-κB signaling by direct targeting IRAK4
Yang et al. [145]	Hemorrhagic	microRNA-223	Lower	Could downregulate NLRP3 to inhibit inflammation and brain edema
Li Y. et al. [146]	Ischemic (MCAO)	miRNA-107	Increased	Might regulate post-stroke angiogenesis and therefore serve as a therapeutic target.
Sun et al. [147]	Ischemic	microRNA-15a/16–1	Increased	Represses pro-angiogenic factors VEGFA and FGF2 and their receptors VEGFR2 and FGFR1
Xin et al. [148]	Ischemic (MCAO)	microRNA-133	Lower	Overexpressing MSCs further stimulates and increases exosomes’ release from astrocytes, possibly by downregulating the RABEPK expression
Xu et al. [149]	Ischemic	microRNA-1906	Increased in glial cells Decreased in neurons	Abolishment of TLR4 protein expression; could ameliorate brain injury in experimental stroke
Iwuchukwu et al. [150]	Hemorrhagic	Panel: miRNA 204-5p + miRNA 9-5p + miRNA-338-3p	Lower	Target: MMP-9 Elevated MMP -> increased damage during acute phase of ICH
Tao Z. et al. [151]	Ischemic	miRNA 99a	Lower	MiR-99a prevented apoptosis and blocked cell cycle progression in neuro-2a cells
Yin et al. [152]	Ischemic	miRNA-497	Increased	miR-497 promotes ischemic neuronal death by negatively regulating anti-apoptotic proteins, bcl-2 and bcl-w
Zhao et al. [153]	Ischemic	miRNA-424	Lower	Expression prevented ischemic brain injury through a mechanism involving suppressing microglia activation
Rahmati et al. [154]	Ischemic	miRNA-210 + HIF1-a	Lower	HIF-1° induces miRNA—210: could prevent apoptosis, protect stem cell survivance and induce angiogenesis
